# Supplementary material for: Pyruvate Dehydrogenase Kinase 1 inhibition mediated oxidative phosphorylation enhancement in cartilage promotes osteoarthritis progression
Source: BMC Musculoskelet Disord. 2023 Jul 20;24:597. doi: 10.1186/s12891-023-06585-6 (PMC10357736; doi:10.1186/s12891-023-06585-6)
Supplement: Supplementary file 1 — Additional file 1. [file 12891_2023_6585_MOESM1_ESM.zip › BP.Gsea.1653623667859/GOBP_ESTABLISHMENT_OF_EPITHELIAL_CELL_POLARITY.html]

Details for gene set GOBP\_ESTABLISHMENT\_OF\_EPITHELIAL\_CELL\_POLARITY[GSEA]

|  || Dataset | 55457\_collapsed\_to\_symbols.cls.cls #OA\_versus\_normal.cls.cls #OA\_versus\_normal\_repos |
| Phenotype | cls.cls#OA\_versus\_normal\_repos |
| Upregulated in class | normal |
| GeneSet | GOBP\_ESTABLISHMENT\_OF\_EPITHELIAL\_CELL\_POLARITY |
| Enrichment Score (ES) | -0.5358172 |
| Normalized Enrichment Score (NES) | -1.7286334 |
| Nominal p-value | 0.0017667845 |
| FDR q-value | 0.1206509 |
| FWER p-Value | 1.0 |
Table: GSEA Results Summary

  

Fig 1: Enrichment plot: GOBP\_ESTABLISHMENT\_OF\_EPITHELIAL\_CELL\_POLARITY      
 Profile of the Running ES Score & Positions of GeneSet Members on the Rank Ordered List

  

| SYMBOL | TITLE | RANK IN GENE LIST | RANK METRIC SCORE | RUNNING ES | CORE ENRICHMENT || 1 | FRMD4B | FERM domain containing 4B [Source:HGNC Symbol;Acc:HGNC:24886] | 1630 | 0.409 | -0.0855 | No |
| 2 | WNT5A | Wnt family member 5A [Source:HGNC Symbol;Acc:HGNC:12784] | 1995 | 0.361 | -0.0774 | No |
| 3 | IFT20 | intraflagellar transport 20 [Source:HGNC Symbol;Acc:HGNC:30989] | 3403 | 0.211 | -0.1655 | No |
| 4 | FERMT1 | FERM domain containing kindlin 1 [Source:HGNC Symbol;Acc:HGNC:15889] | 3687 | 0.186 | -0.1687 | No |
| 5 | EZR | ezrin [Source:HGNC Symbol;Acc:HGNC:12691] | 3817 | 0.174 | -0.1612 | No |
| 6 | SLC9A3R1 | SLC9A3 regulator 1 [Source:HGNC Symbol;Acc:HGNC:11075] | 4111 | 0.145 | -0.1694 | No |
| 7 | GOLPH3 | golgi phosphoprotein 3 [Source:HGNC Symbol;Acc:HGNC:15452] | 4420 | 0.119 | -0.1813 | No |
| 8 | MSN | moesin [Source:HGNC Symbol;Acc:HGNC:7373] | 6919 | -0.074 | -0.3680 | No |
| 9 | CLASP1 | cytoplasmic linker associated protein 1 [Source:HGNC Symbol;Acc:HGNC:17088] | 7151 | -0.092 | -0.3766 | No |
| 10 | PTK7 | protein tyrosine kinase 7 (inactive) [Source:HGNC Symbol;Acc:HGNC:9618] | 7725 | -0.136 | -0.4075 | No |
| 11 | RHOA | ras homolog family member A [Source:HGNC Symbol;Acc:HGNC:667] | 8601 | -0.201 | -0.4552 | No |
| 12 | TCF15 | transcription factor 15 [Source:HGNC Symbol;Acc:HGNC:11627] | 8925 | -0.226 | -0.4576 | No |
| 13 | FOXF1 | forkhead box F1 [Source:HGNC Symbol;Acc:HGNC:3809] | 9933 | -0.315 | -0.5042 | Yes |
| 14 | SH3BP1 | SH3 domain binding protein 1 [Source:HGNC Symbol;Acc:HGNC:10824] | 10016 | -0.323 | -0.4781 | Yes |
| 15 | FAT1 | FAT atypical cadherin 1 [Source:HGNC Symbol;Acc:HGNC:3595] | 10318 | -0.353 | -0.4660 | Yes |
| 16 | SIPA1L3 | signal induced proliferation associated 1 like 3 [Source:HGNC Symbol;Acc:HGNC:23801] | 11036 | -0.435 | -0.4780 | Yes |
| 17 | ARF6 | ADP ribosylation factor 6 [Source:HGNC Symbol;Acc:HGNC:659] | 11084 | -0.442 | -0.4372 | Yes |
| 18 | RAP2A | "RAP2A, member of RAS oncogene family [Source:HGNC Symbol;Acc:HGNC:9861]" | 11179 | -0.454 | -0.3989 | Yes |
| 19 | CYTH1 | cytohesin 1 [Source:HGNC Symbol;Acc:HGNC:9501] | 11804 | -0.550 | -0.3920 | Yes |
| 20 | MYO9A | myosin IXA [Source:HGNC Symbol;Acc:HGNC:7608] | 12166 | -0.635 | -0.3563 | Yes |
| 21 | PARD3 | par-3 family cell polarity regulator [Source:HGNC Symbol;Acc:HGNC:16051] | 12489 | -0.743 | -0.3066 | Yes |
| 22 | CYTH3 | cytohesin 3 [Source:HGNC Symbol;Acc:HGNC:9504] | 12632 | -0.814 | -0.2358 | Yes |
| 23 | HES1 | hes family bHLH transcription factor 1 [Source:HGNC Symbol;Acc:HGNC:5192] | 12676 | -0.849 | -0.1538 | Yes |
| 24 | FRMD4A | FERM domain containing 4A [Source:HGNC Symbol;Acc:HGNC:25491] | 12679 | -0.850 | -0.0686 | Yes |
| 25 | CDC42 | cell division cycle 42 [Source:HGNC Symbol;Acc:HGNC:1736] | 12681 | -0.852 | 0.0170 | Yes |
Table: GSEA details [plain text format]

  

Fig 2: GOBP\_ESTABLISHMENT\_OF\_EPITHELIAL\_CELL\_POLARITY      
 Blue-Pink O' Gram in the Space of the Analyzed GeneSet

  

Fig 3: GOBP\_ESTABLISHMENT\_OF\_EPITHELIAL\_CELL\_POLARITY: Random ES distribution      
 Gene set null distribution of ES for **GOBP\_ESTABLISHMENT\_OF\_EPITHELIAL\_CELL\_POLARITY**
